# Tissue Expression Of *LPHN3* in Breast Cancer: An Immunohistochemistry Method

**DOI:** 10.31557/APJCP.2020.21.11.3339

**Published:** 2020-11

**Authors:** Kwuntida Uthaisar Kotepui, Manas Kotepui, Duangjai Piwkham, Apiram Songsri, Lek Charoenkijkajorn, Tidamas Kongnok, Yupaporn Chanil

**Affiliations:** 1 *Medical Technology Program, School of Allied Health Sciences, Walailak University, Nakhon Si Thammarat 80161, Thailand. *; 2 *Department of Pathology, Hatyai Hospital, Songkhla 90110, Thailand. *

**Keywords:** LPHN3, breast cancer, lymph node metastasis

## Abstract

**Objective:**

Breast cancer is one of the most important public health problems among women worldwide. It is a major cause of morbidity especially among women in developing countries including Thailand. The purpose of this study was to study the expression of LPHN3 protein in normal breast tissue compared to breast cancer tissue. Methods: We had studied the expression of LPHN3 in 65 breast tissues using an immunohistochemistry method. The association between *LPHN3* expression and breast cancer metastasis to nearby axillary lymph nodes was also examined.

**Results:**

Among the 65 breast cancer and normal breast tissues examined, *LPHN3* expression with an immunohistochemistry index (IHC index) greater than 4 was more frequently found in breast cancer tissues than in normal breast tissues (P-value = 0.001, OR (95% CI) = 7.04 (2.16-23)). Moreover, a high expression of *LPHN3* (IHC index > 4) was more frequently found in breast cancer tissues with negative axillary lymph nodes than in those with positive ones (P-value = 0.038, OR (95% CI) = 0.25 (0.07-0.96)). LPHN3 protein might be a new metastasis suppressor gene in breast cancer and a marker for breast cancer metastasis prevention.

**Conclusions:**

The present study indicated that a decrease of LPHN3 protein expression in breast cancer tissue might be a marker indicating the aggressiveness of breast cancer. These results also suggested that a decrease of *LPHN3* expression could be functionally involved in breast cancer progression and metastasis.

## Introduction

Breast cancer is the most common cancer among women worldwide affecting 2.1 million persons annually, and it is the leading cause of cancer death among women (WHO). In Thailand, breast cancer is the third most common cancer with 19,510 new cases in 2018 of which 5,902 cases resulted in death (Bray et al., 2018). The data from a study at Songklanagarind Hospital in Southern Thailand estimated 671 breast cancer cases in 2018 (Prechawittayakul, 2009). Breast cancer is still frequently diagnosed at an advanced stage, and the study of protein expression as a biomarker is still elusive. Therefore, a better understanding of the molecular regulation involving breast cancer progression may help to discover effective molecular markers to evaluate diagnosis and prognosis. These will help increase the success rate of therapy with lower mortality (Wang, 2017) because tumor metastasis can lead to poor chances of survival for patients (Jemal et al., 2008). An infiltrating duct carcinoma with regional lymph node metastasis is the most common breast cancer type among Thai patients (Kotepui and Chupeerach, 2014). Metastasis is the final progression of solid cancer. This involves tumor cell intravasation, circulation, extravasation, angiogenesis, and continued growth in other organs and tissues (Valastyan and Weinberg, 2011). The majority of cancers begin developing metastatic clones and spreading via lymphatic vessels to other lymph nodes and other organs. The detection of tumor with lymph node metastases contribute to major prognostic implications and the selection of adjuvant therapies for improving patient survival (Wu et al., 2014).

LPHN3 (the latrophilin 3 gene) is a member of the G-protein coupled receptor (GPCR) family with a large extracellular and intracellular domain, containing several cell adhesion modules such as cadherin, IgG, laminin A, thrombospondin type 1, galactose lectin, EGF, and transmembrane segments that may be involved in intracellular signaling during cell-to-cell adhesion (Wu et al., 2014). A previous study indicated that *LPHN3* was up-regulated significantly in a transgenic mice model that over-expressed myocilin (Paper et al., 2008). Altered *LPHN3* expressions in brain ischemia have been observed (Bin Sun et al., 2002). Mice lacking the *LPHN3* expression resulted in attention deficit-hyperactivity disorder (ADHD), the most common psychiatric disorder in childhood and adolescence (Wallis et al., 2012). Nevertheless, the study of *LPHN3* in human cancer is poorly understood.

Increased mRNA expression of *LPHN3* and *MMP13 *was significantly associated with axillary node metastasis assessed by RT-PCR (Kotepui et al., 2012). However, the *LPHN3* expression at the protein level in breast cancer is still unelucidated. The present study aimed to evaluate the *LPHN3* expression in breast cancer. Moreover, *LPHN3* expression related to axillary lymph node metastasis was also examined.

## Materials and Methods

*Human subjects and tissue specimens*

Tissue samples, including invasive ductal breast cancer and normal breast tissues that were diagnosed and surgically treated, were obtained from the Department of Pathology, Hatyai Hospital, Songkhla Province, between January to December 2017. The female patients had not received prior radiotherapy or neoadjuvant therapies before recruiting the tissues. The patient characteristics include age at diagnosis, type of tissue, grade of tumor, and regional lymph node status. Grading standard was commonly used to assign the scores of histological grades of breast cancer. Grade I is well-differentiated tumors, Grade II is moderately differentiated tumors, and Grade III is poorly differentiated tumors. This study was performed under a protocol approved by the Ethic Committee of Hatyai Hospital and the Ethical Clearance Committee on Human Rights Related to Researches Involving (WU-EC-MT-2-045/59). Informed consent was not obtained from participants, but patient records/information was anonymized and de-identified prior to this analysis. The name and Hospital Number (HN) of patients were not revealed.

*Immunohistochemistry (IHC)*

*LPHN3* was detected using standard immunohistochemistry protocols. Specifically, the paraffin sections were deparaffinized and hydrated, and then the endogenous peroxidase was blocked with H_2_O_2_. After blocking with normal serum, the sections were incubated with 1:100 of *LPHN3* Polyclonal Antibody (ab150794, Thermo Fisher Scientific Inc, MA, USA) at room temperature for overnight, and secondary Ab and peroxidase activity was visualized with a diaminobenzidine (DAB) solution. The frequency of *LPHN3* positive cells was semi-quantitatively scored on the basis of the percentage of positive cells, where 0% = negative, 1-25% = +1, 26-50% = +2, and > 50% = +3. The intensity of the *LPHN3* expression was scored as weak = 1, moderate = 2, and strong = 3. The average *LPHN3* expression of each section was calculated as intensity multiplied by frequency and categorized as low (≤ 4) or high (> 4).

*Statistical analysis*

Statistical analyses were performed using SPSS Statistics for Windows, Version 17.0 (SPSS Inc., Chicago, IL, USA), and the computer program Prism (GraphPad Software, La Jolla, CA). Student’s t-test was used for comparison between two groups. A P-value of < 0.05 was considered statistically significant. *LPHN3* expression was evaluated for association with clinicopathological findings using the Chi2-test (*P < 0.05, **P < 0.01, and ***P < 0.001).

## Results

*Characteristics of included breast cancer cases*

Among the 65 breast tissues retrieved from Hatyai Hospital, 22 cases (33.8%) were normal breast tissues whereas 43 cases (66.2%) were breast cancer tissues. Among the 43 breast cancer tissues, 18 cases (41.9%) were positive axillary lymph nodes whereas 25 cases (58.1%) were negative axillary lymph nodes. The mean age of patients was 46.8±3.04 years with an average tumor size of 3.04±1.55 centimeters. Most of the breast cancer cases were Grade II (23 cases, 59%) and Grade III (12 cases, 30.8%). All patient characteristics are shown in Table 1.

*Immunohistochemical expression of LPHN3 in normal and breast cancer tissues*

**Figure 1:**
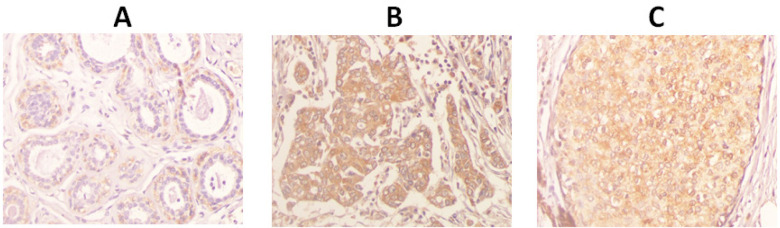
Immunohistochemistry of Normal Breast Tissue (A), breast cancer tissue with negative lymph nodes (B), and breast cancer tissue with positive lymph nodes (C) (40X)

The *LPHN3* expression in all the types of breast tissues is shown in Figure 1 and Table 2. The results showed that most of the normal breast cancer tissues exhibited an IHC index at 4 (7 cases, 31.8%). Most of the breast cancer tissues with negative axillary lymph nodes exhibited an IHC index at 6 (15 cases, 60%). Most of the breast cancer tissues with positive axillary lymph nodes also exhibited an IHC index at 6 (7 cases, 38.9%). 

*Prognosis factor of LPHN3 expression in breast cancer tissues with positive axillary lymph nodes*

The IHC index was categorized into > 4 or ≤ 4 based upon a mean IHC index (4.8). *LPHN3* expression with an IHC index > 4 was more frequently found in breast cancer tissues than in normal tissues (P-value = 0.001, OR (95% CI) = 7.04 (2.16-23)). Moreover, *LPHN3* expression with an IHC index > 4 was more frequently found in breast cancer tissues with negative axillary lymph nodes than in those with positive ones (P-value = 0.038, OR (95% CI) = 0.25 (0.07-0.96)) (Table 3). Regression analysis of the *LPHN3* expression with an IHC index > 4 indicated significance of *LPHN3* expression as both a breast cancer prevention biomarker and also for anti-tumor metastasis to axillary lymph nodes (P-value = 0.001) (Table 4). 

**Table 1:** Patient Characteristics Included in the Study

Patient characteristics	Frequency (%)
Age (mean±SD)	46.8±3.04
Breast tissue	
Normal	22 (33.8)
Cancer	43 (66.2)
Tumor size (mean±SD)	3.04±1.55
Grade	
Grade I	4 (10.3)
Grade II	23 (59)
Grade III	12 (30.8)
Lymph node status	
Negative	25 (58.1)
Positive	18 (41.9)

**Table 2:** *LPHN3 *Expression in All the Types of Breast Tissues

IHC index	Types of breast tissues			
	Normal (%)	Breast cancer with negative axillary lymph nodes (%)	Breast cancer with positive axillary lymph nodes (%)	Total (%)
1	4 (18.2)	1 (4)	2 (11.1)	7 (10.8)
2	3 (13.6)	1 (4)	3 (16.7)	7 (10.8)
3	3 (13.6)	0	1 (5.6)	4 (6.2)
4	7 (31.8)	3 (12)	3 (16.7)	13 (20)
6	3 (13.6)	15 (60)	7(38.9)	25 (38.5)
9	2 (9.1)	5 (20)	2 (11.1)	9 (13.8)

P-value by Pearson Chi-Square

**Table 3:** IHC Index cutoff of *LPHN3* Expression (> 4 vs ≤ 4) in All the Types of Breast Tissues

LPHN3 expression	Breast tissues		P-value	OR (95% CI)
	Normal (%)	Cancer (%)		
IHC index				
≤ 4	17 (73.3)	14 (32.6)	0.001	7.04 (2.16-23)
> 4	5 (22.7)	29 (67.4)		
	Axillary lymph nodes			
	Negative (%)	Positive (%)		
IHC index				
≤ 4	5 (20)	9 (50)	0.038	0.25 (0.07-0.96)
> 4	20 (80)	9 (50)		

P-value by Pearson Chi-Square

**Table 4:** Regression Analysis of *LPHN3 *Expression with IHC Index > 4

	B	S.E.	Wald	df	Sig.	Exp(B)
*LPHN3* expression as a breast cancer marker						
IHC index	-1.952	0.604	10.447	1	0.001	0.142
Constant	1.758	0.484	13.178	1	0	5.8
*LPHN3* expression as a breast cancer marker for tumor metastasis to axillary lymph nodes						
IHC index	1.386	0.687	4.07	1	0.044	4
Constant	-0.799	0.401	3.958	1	0.047	0.45

## Discussion

The present study evaluated the *LPHN3* expression in normal breast and cancer tissues. The results revealed that *LPHN3* was generally expressed in both normal breast and cancer tissues with different intensities. *LPHN3* was more highly expressed in breast cancer tissues than in normal breast tissues. However, *LPHN3* expression was decreased in breast cancer tissues with positive axillary lymph nodes when compared with negative axillary lymph nodes.

*LPHN3* is a brain-specific member of the G-protein coupled receptor family associated with both ADHD genetic susceptibility and methylphenidate pharmacogenetics (Bruxel et al., 2015). High *LPHN3* expression has been previously reported in a transgenic mice model that over-expressed myocilin and in mice after brain ischemia (Wu et al., 2014; Paper et al., 2008). Mice lacking the *LPHN3* expression resulted in attention deficit-hyperactivity disorder (ADHD), the most common psychiatric disorder in childhood and adolescence (Wallis et al., 2012). It has also been demonstrated that *LPHN3* activation in pancreatic islets reduces insulin secretion (Rothe J et al., 2019).

The increase of *LPHN3* in breast cancer tissues was at significantly higher levels when compared to healthy breast tissues. It has been previously reported that primary breast tumors and MCF-7 cells expressed comparable amounts of *LPHN3* (Yasinska et al., 2019). Increased mRNA expression of *LPHN3* and *MMP13* was significantly associated with axillary node metastasis assessed by RT-PCR (Kotepui et al., 2012). In addition, Jahn et al., (2016) found 1% mutation of *LPHN3* in the usual ductal hyperplasia (UDH) of the breast. In contrast, our study revealed that the *LPHN3* expression was more frequently found in breast cancer tissues with negative axillary lymph nodes than in those with positive ones. 

In Thailand, the incidence rate of breast cancer varies geographically based upon diverse lifestyles, behaviors, and risk profiles of the northern, northeastern, central, and southern regions of Thailand (Jordan et al., 2009). For example, a previous study showed that tumors among Muslims were histologically homogeneous; whereas tumors among Buddhists exhibited heterogeneity, which may have genetic, biological, and management implications (Pang et al., 2018). 

The intensity of *LPHN3* immuno-reactivity was different according to the histological grading subtype of breast cancer. *LPHN3* was highly expressed in moderately to poorly-differentiated carcinomas but down-regulated in well-differentiated tumors. Axillary lymph nodes are the most common initial site of metastatic disease (Woods et al., 2019). It is not only a marker of diagnosis at a later point in the natural history of breast cancer but also a marker of an aggressive phenotype (Jatoi et al., 1999). Although the high expression of this protein was found in poor differentiation histological grade (8/12, 66.7%), the lower expression of this protein was also found in well differentiation histological grade (4/12, 33.3%). Nevertheless, the statistically significance of lower *LPHN3* expression was not statistically significance (p=0.053). This might due to the low sample size evaluated in the present study. In this study, the association of low *LPHN3* expression and lymphatic invasion was observed. Moreover, this study found that higher *LPHN3* expression was not related to axillary lymph node metastasis.

In conclusion, our findings indicated that the decrease of the LPHN3 protein expression in breast cancer tissues may be a new important tumor marker and a new marker indicating aggressiveness of breast cancer. These results also suggested that *LPHN3* could be functionally involved in breast cancer progression and metastasis. However, further studies need to examine the details of the underlying mechanism of low LPHN3 protein expression, which may influence the breast cancer metastasis phenotype.
